# Detection and drivers of exposure and effects of pharmaceuticals in higher vertebrates

**DOI:** 10.1098/rstb.2013.0570

**Published:** 2014-11-19

**Authors:** Richard F. Shore, Mark A. Taggart, Judit Smits, Rafael Mateo, Ngaio L. Richards, Steve Fryday

**Affiliations:** 1 Centre for Ecology & Hydrology, Lancaster Environment Centre, Library Avenue, Bailrigg, Lancaster LA1 4AP, UK; 2 Environmental Research Institute, University of the Highlands and Islands, Castle Street, Thurso, Caithness KW14 7JD, UK; 3 Department of Ecosystem and Public Health, Faculty of Veterinary Medicine, University of Calgary, 3280 Hospital Drive NW, Calgary, Alberta, Canada AB T2N 4Z6; 4 Instituto de Investigación en Recursos Cinegéticos, IREC (CSIC, UCLM, JCCM), Ronda de Toledo s/n, 13005 Ciudad Real, Spain; 5 Working Dogs for Conservation, 52 Eustis Road, Three Forks, MT 59752, USA; 6 Food and Environment Research Agency (FERA), Sand Hutton, York YO41 1LZ, UK

**Keywords:** ecopharmacovigilance, wildlife toxicology, environmental monitoring, emerging contaminants, sentinel species, risk assessment

## Abstract

Pharmaceuticals are highly bioactive compounds now known to be widespread environmental contaminants. However, research regarding exposure and possible effects in non-target higher vertebrate wildlife remains scarce. The fate and behaviour of most pharmaceuticals entering our environment via numerous pathways remain poorly characterized, and hence our conception and understanding of the risks posed to wild animals is equally constrained. The recent decimation of Asian vulture populations owing to a pharmaceutical (diclofenac) offers a notable example, because the exposure route (livestock carcasses) and the acute toxicity observed were completely unexpected. This case not only highlights the need for further research, but also the wider requirement for more considered and comprehensive ‘ecopharmacovigilance’. We discuss known and potential high risk sources and pathways in terrestrial and freshwater ecosystems where pharmaceutical exposure in higher vertebrate wildlife, principally birds and mammals, may occur. We examine whether approaches taken within existing surveillance schemes (that commonly target established classes of persistent or bioaccumulative contaminants) and the risk assessment approaches currently used for pesticides are relevant to pharmaceuticals, and we highlight where new approaches may be required to assess pharmaceutical-related risk.

## 1. Introduction

In line with the expansion and changing age structure of the human population, total global spending on human medicine has increased by approximately $50 Bn yr^−1^ since 2007, and will reach approximately $1.2 Tn by 2017 [1]. Large developing nations, such as China, India and Brazil, are promoting swift growth in their pharmaceutical manufacturing, human healthcare [1] and intensive agricultural sectors. Increasing use of pharmaceuticals in human and veterinary medicine has led to a concurrent rise in ‘medicated’ discharges to the environment and this presents a significant challenge to risk assessors and regulators charged with environmental protection. A wide range of drugs have now been detected in multiple environmental compartments globally [2–6].

It can be argued that the likelihood of pharmaceuticals causing widespread acute effects in wild higher vertebrates is low. This is because extensive safety testing is conducted during the development of modern human and veterinary drugs and also because of the existence of regulatory and environmental protection frameworks. However, a number of significant weaknesses remain with respect to assessment of risk to wild vertebrates. Although there is considerable testing of pharmaceuticals on some mammal species, there is less on birds and data on pharmacokinetics and therapeutic doses in birds are limited. What data there are suggest that there can be substantial differences in sensitivity and toxicity between birds and mammals and, in fact, between different mammal species [7–10]. In addition, the potential for bioaccumulation or bioconcentration and resultant elevated exposure in higher wildlife is not well characterized, particularly for terrestrial systems [11,12]. Risk assessments also focus on single pharmaceuticals, whereas wildlife will typically be exposed to complex mixtures, and our understanding of how pharmaceuticals interact with each other and affect toxicity is limited [13,14]. Finally, regulatory frameworks that monitor and restrict pharmaceutical emissions to the environment are often absent or ineffectual in many developing countries [15] and, even when in place, there may be a need to upgrade treatment processes (at sewage treatment plants (STPs) for example) to adequately curtail emissions of pharmaceuticals to the environment [16,17].

Significant adverse effects in terrestrial and aquatic organisms caused by pharmaceuticals have demonstrated inadequacies in current risk assessment and regulatory processes. Two of the most notable cases that have caused global concern are (i) the near extirpation of *Gyps* vulture populations in large parts of Asia following exposure to non-steroidal anti-inflammatory drugs (NSAIDs) [18–20] and (ii) the feminization of wild male fish from exposure to synthetic oestrogen 17α-ethynyloestradiol (EE_2_), a common ingredient in the human contraceptive pill [21,22], although it is not known whether such endocrine effects likewise occur in higher aquatic vertebrates. Furthermore, pharmaceuticals are designed to be highly bioactive, often for prolonged periods at low doses. Therefore, although severe adverse effects on wildlife have been detected, any effects of pharmaceuticals on wild higher vertebrates are more likely to be chronic and subtle. Such effects will be difficult to detect and quantify or to attribute to a particular input. However, even basic monitoring data on exposure and effects of pharmaceuticals in higher wildlife are scarce [23].

Here, we review the current state of knowledge regarding pharmaceutical contamination and effects in wild higher vertebrates, specifically birds and mammals. There are few data for birds and mammals but, given the severe impact of diclofenac on Asian vultures, our aim is to draw attention to potentially relevant exposure pathways. We examine which exposure-mediating factors are incorporated in current risk assessment processes and consider how exposure of wild higher vertebrates to pharmaceuticals may best be detected. The question underpinning this review is: can the current systems used to detect exposure and assess risks to higher vertebrates from chemicals (such as pesticides) be applied to pharmaceuticals? Unless relevant to specific food chain pathways (i.e. for piscivorous species), we do not consider exposure in wild fish as this is covered comprehensively elsewhere [4,13,21,22,24,25], nor do we review laboratory-based exposures aimed at detecting specific compound–species effects. Instead, we focus on environmentally relevant field exposure situations, primarily for birds and mammals, in freshwater and particularly terrestrial habitats worldwide.

## 2. Key exposure sources and pathways

**Figure 1.**
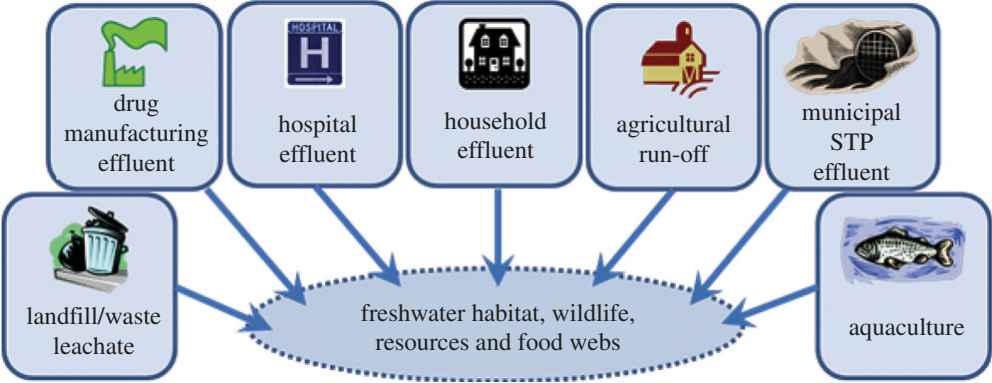
Pathways of pharmaceutical releases to freshwaters. (Online version in colour.)

Concern over the release of pharmaceuticals to the environment has, until recently, focused on freshwaters, reflecting the fact that human medicines almost invariably end up in aquatic systems because of urinary and faecal excretion of prescription and recreational drugs, and improper disposal of old medication [26]. Pharmaceuticals can enter freshwater systems through a number of pathways (figure 1), the principal routes being the discharge of liquid waste (domestic sewage, hospital or industrial effluent) into aquatic habitats [16,27]. Research has tended to centre on municipal STPs [16,28,29], principally in Europe and North America, on hospitals [28,30] and on drug manufacturing facilities [15,29,31] which emit less volume but more concentrated effluent. Aquaculture has also attracted attention as a source of pharmaceutical inputs to freshwaters [32,33].

Untreated domestic, municipal and industrial sewage effluent containing drugs can also enter freshwater via misconnections, failed sewage pipework, combined sewer overflows, septic tanks, or owing to a complete absence of sewer and STP infrastructure. Recent data suggest that even in high-income countries about 30% of wastewater is discharged untreated, a figure that increases to about 92% in low-income countries [34,35]. The standards for solid waste management likewise vary considerably around the world [36]. Pharmaceuticals are now a known component of landfill leachate and can affect groundwater quality [37], but the wider impacts of leachate on freshwater resources and food webs have not been investigated. Landfill engineering is usually absent in most low-income countries and disposal sites are simply open dumps, with little to no leachate control or mitigation. In contrast, sanitary landfills in other regions (i.e. in Europe, North and South America) may be fully lined and engineered to prevent leachate impacting freshwater resources [36]; leachate treatment plants may also be used.

In terms of risk from pharmaceuticals, the terrestrial habitat has received far less attention compared with freshwaters. In general, terrestrial inputs exist where liquid or solid waste contaminated with drugs is deposited on land (table 1). Point sources of emissions include carcass dumps and landfill sites as well as STPs and other wastewater holding areas/lagoons [38] that may provide habitat or substrate for the prey of terrestrial predators. Agriculture is the other major source by which pharmaceuticals are likely to be emitted to the terrestrial environment, although inputs may lead to simultaneous contamination of freshwater habitats via surface run-off and groundwater leaching [39–41]. Industrial agricultural systems, such as concentrated animal feeding operations (CAFOs), undertake confined, highly intensive rearing of animals in areas without vegetation (indoors on concrete or outdoors on un-vegetated feedlots) and are an increasingly common means in the USA and beyond of meeting global demand for food. The operations rely heavily on the constant administration of veterinary medication (antibiotics, steroids, growth promoters and antiparasitics) and may represent important sources of environmental contamination [42–44]. More diffuse environmental inputs may arise from fertilization of agricultural land with manure, slurry, biosolids and STP effluent. Non-intensive livestock rearing may likewise result in diffuse emissions through processes such as direct excretion of veterinary products by animals, shedding/moulting of fur, feathers and other similar material that may contain residues of veterinary products, and through the death of medicated livestock in areas where carcasses remain un-retrieved.

**Table 1.** Potential terrestrial exposure pathways associated with different processes/practices.

process/practice	possible exposure route/risk
landfill/municipal solid waste disposal *disposal of waste medication*	— direct ingestion of medication/leachate by scavengers — indirect ingestion by insectivores (e.g. birds/bats) consuming insects feeding on waste/leachate — contamination of local freshwater resources and land by leachate containing drugs
animal waste disposal *disposal of ‘medicated’ animal carcasses*	— carcass may contain medication administered before death—ingestion by scavengers (or carnivores) — indirect ingestion by insectivores (e.g. birds/bats) consuming insects feeding on medicated carcasses — sustained release bolus (long duration medication—as tablets) may be present within carcass digestive tract
liquid waste processing *STP treatment of ‘medicated’ effluent*	— STPs ‘attract’ certain species, e.g. insectivores or aquatic birds at trickling filters or tertiary treatment/polishing lagoons (respectively) — direct ingestion of wastewater and/or long-term dermal exposure in lagoons (aquatic birds and mammals) — indirect ingestion by insectivores (e.g. birds/bats) consuming invertebrates feeding on effluent (e.g. at trickling filters) or emerging from lagoons — potential bioaccumulation and food chain/trophic transfer within lagoon ecosystems
applying manure, slurry or STP biosolids/effluent to land *re-use of ‘medicated’ waste in agriculture*	— potential for persistence and/or bioaccumulation of certain compounds in soil and soil invertebrates — food chain/trophic transfer in agricultural ecosystems — direct ingestion by birds/mammals — indirect ingestion by insectivores (e.g. birds/bats) consuming soil/aerial invertebrates
livestock/poultry production *‘medicated’ faeces deposition*	— livestock/poultry receiving medication generate contaminated faeces and urine — direct ingestion by soil and aerial invertebrates — indirect ingestion by insectivores (birds/mammals, etc.) — contact with residues in hair, wool, feathers and other material used as nesting or ingestion of this material — direct ingestion by coprophagous vertebrates — risk of high exposure if sustained release bolus (long duration medication—as tablets) in use — intensive outdoor operations (e.g. feedlot CAFOs) may pose a particularly elevated risk

Exposure pathways for higher vertebrates may be directly through ingestion of and contact with contaminated water, through herbivory of contaminated forage, secondary exposure through predation of contaminated invertebrates and lower vertebrates, and direct exposure through coprophagy. While the potential impact of pharmaceuticals on invertebrates, particularly coprophagous insect communities, has received intense scrutiny [45–47], the exposure risks to higher vertebrates within food webs largely remain to be investigated [48]. For aquatic birds and mammals, foraging around STPs is arguably the most important exposure route. Potential exposure routes for terrestrial higher vertebrates are perhaps more diverse and summarized in table 1.

## 3. Factors considered in risk assessment that mediate exposure and effect

In general, there is lack of guidance when considering the key factors affecting the exposure of wildlife to pharmaceuticals. Given this, it may be useful to consider the existing, very well developed, approaches used for estimating exposure of birds and mammals to another group of anthropogenic compounds, plant protection products (PPPs). In Europe, regulatory approaches for estimating acute and chronic risks to birds and mammals from PPPs involve calculating toxicity : exposure ratios (TERs). These estimate how much the toxic dose (for example, an LD_50_) exceeds the estimated exposure [49]. Broadly equivalent approaches are used elsewhere and for other chemicals, although they may use other ratios such as a risk characterization ratio (RCR)—essentially, the inverse of a TER. A major factor when calculating a TER or RCR is estimating the exposure term. This involves accounting for various factors that may modify the uptake of PPPs by organisms. These are considered here to examine whether similar factors are equally likely to modify the risk of exposure to pharmaceuticals.

It is currently assumed that the only significant exposure route for PPPs in birds and mammals is through ingestion (oral exposure). Risk assessments are tiered, and an initial screening tier uses relevant acute or chronic toxicity data, ‘indicator’ species and worst-case estimates of exposure. If a PPP fails the screening, first tier assessments are conducted using ‘generic focal species’ and more realistic estimations of dietary exposure for acute and reproductive risk. These include standardized approaches to assess risk from endocrine-disrupting chemicals (EDCs), from metabolites, and from bioaccumulation; three bioaccumulation food-chain scenarios are used (earthworm to earthworm-eating birds and mammals; fish to piscivorous bird/mammal; biomagnification in food chains). Application of such approaches may be particularly important for pharmaceuticals as the majority are ionizable and, by their nature, readily metabolizable. Subsequent higher test tiers for PPPs use specific focal species and typically employ a range of refinement options tailored towards realistic exposures for those species. Toxicity endpoints can also be refined. For example, phase-specific reproductive endpoints can be used to relate timing of exposure to that of reproductive cycles in birds and mammals [50,51].

While the general framework for assessing PPP risk may be applicable to pharmaceuticals, some of the refinements used to reduce exposure from worst case to more realistic values may not always be appropriate. Such refinements include (i) estimating food intake rate for focal species based on allometric equations of daily energy expenditure (DEE) and water flux [52,53], (ii) bioaccumulation, and (iii) the fact that only a proportion of ingested food is contaminated. Some scavengers, for example, actively seek and feed communally on carcasses and so it is possible that they predominantly feed on sick and medicated animals. Hence refinement of risk based on an assumption that only a proportion of ingested food is contaminated may not be appropriate. Scavengers also gorge at carcasses and can eat more at one feed than would be predicted just from estimates of intake based on DEE requirements [54]. Reliance on such estimates may therefore underestimate exposure. Scavengers may also be selective in terms of the tissues they consume. Preferential feeding on stomach contents and soft tissues such as liver, kidney and heart is likely to increase exposure and risk [55] and may occur at multiple trophic levels, thereby enhancing bioaccumulation along food chains. Clearly, oral exposure risk assessments need to account for the pharmacokinetics of the compound, although for pharmaceuticals even the most basic parameters such as half-lives are inadequately characterized in higher vertebrate wildlife species. Risk assessments also need to consider the feeding preferences and general ecology of the species of interest because these may alter and even enhance exposure risk. This is true for PPPs as well as for pharmaceuticals, although refinement stages for PPPs are usually predicated on the expectation that they will reduce (rather than enhance) estimated risk.

PPP risk assessments for birds and mammals do not currently account for dermal or inhalation exposure, although it has been argued that dermal uptake may sometimes be a significant exposure route [56], and the potential for inhalation during spray applications is clear [57]. Dermal exposure to PPPs, and equally pharmaceuticals, may arise from contact with contaminated water, while moving across contaminated soil or while dust/puddle bathing. Species such as small mammals, that not only forage on contaminated soil but also burrow into it, may be at particular risk, as may aquatic vertebrates that come into contact with contaminated water. The potential for inhalation exposure of pharmaceuticals by higher wildlife is less obvious, except perhaps for dust inhalation associated with treatments in intensive livestock rearing [58]. The Environmental Protection Agency in the USA is developing models for assessing PPP exposure via both dermal and inhalation routes in birds [59] and these may prove a useful starting point should either or both routes be considered important for pharmaceuticals.

As well as considering whether approaches used in PPP exposure assessments can be applied to pharmaceuticals, it is also relevant to ask whether the TER toxicity endpoints are appropriate when assessing risks from pharmaceuticals. Current toxicity assessments used in PPP risk assessments for birds and mammals use endpoints that are directly related to mortality and reproduction. These are obviously two key parameters that directly impact population dynamics and can be assessed to some extent in laboratory tests. However, the current range of toxicity endpoints used for PPPs has been criticized as too limited for birds but overabundant and too detailed for mammals; the consequence is that it is often difficult to extrapolate realistically from the laboratory to the field [60]. If pharmaceuticals are indeed most likely to have subtle physiological, immunological, behavioural and neurological effects in wild higher vertebrates, such effects could have both direct and indirect impacts on long-term survival and on behavioural phases of the reproductive cycle. Some or possibly many such effects may commonly go undetected using the standardized PPP tests currently undertaken in artificially controlled conditions on laboratory-bred species. There may well be a need to review likely effects associated with the most prevalent classes of pharmaceuticals released to the environment and to develop new toxicity endpoints that are both appropriate to detecting such effects and are relevant in terms of impacting populations. Any such developments would be equally likely to increase the robustness of risk assessments for PPPs and all other chemicals.

## 4. Evidence of exposure and effects of pharmaceuticals on higher wildlife

Few field data have been gathered on exposure to, and effects of, pharmaceuticals in higher vertebrate wildlife. The studies conducted to date can be broadly separated as those considering impacts associated with (i) STPs and freshwater, and (ii) terrestrial systems.

### (a) Impacts on higher vertebrates associated with waste water irrigation and sewage treatment plants

The use of sewage water for irrigation poses a potential risk to wildlife. We are not aware of specific studies on wild higher vertebrates but there have been incidences of hyperoestrogenism in cattle fed alfalfa that was irrigated with untreated sewage water containing phytoestrogens [61]. Interestingly, studies on alfalfa that was irrigated with water containing oestrogenic contaminants (17β-oestradiol and oestrone) concluded that alfalfa growth was increased when irrigated with water containing lower oestrogen concentrations and suppressed when the water contained high concentrations [62]. This suggests that the effects of oestrogens can and do cross between plants and animals, but no details were discussed on the concentrations of oestrogens that resulted in variable growth of the alfalfa, or on the amounts of affected alfalfa that were consumed by the cattle.

Several studies have now investigated risks to birds feeding on invertebrates exposed to STP effluent at trickling filters. These have indicated that invertebrates living on and emerging from STP trickling filters have the potential to bioaccumulate/bioconcentrate certain pharmaceutical EDCs [63,64]. Adult male starlings (*Sturnus vulgaris*) experimentally exposed to environmentally relevant levels of various EDCs displayed altered immune function and changed development that affected behaviours such as singing [65]; reduced growth and depressed immunocompetence were also observed in the nestlings [66]. Additional species of vertebrate wildlife likely to be exposed through the food web include those that take insects on the wing (such as swallows (Hirundinidae), swifts (Apodidae) and bats (Chiroptera)). The population-level consequences of such exposure, and the specific role played by synthetic pharmaceuticals as opposed to natural and non-pharmaceutical-EDCs, remain to be elucidated.

With regard to general contamination of freshwater habitats, informative data are now available on the impacts of certain pharmaceuticals and pharmaceutical-EDCs, such as EE_2_, in some species, particularly fish [22,67–69]. Published research on exposure and effects in mammals or birds that share the same freshwater habitat or that could feed on medicated prey is, however, extremely rare. A study to evaluate bioaccumulation potential of pharmaceuticals in the diet of ospreys (*Pandion haliaetus*), a well-known sentinel of environmental pollution, has been reported to date as a conference abstract [70]. This study used an integrated modelling approach to estimate potential pharmaceutical doses to nestling ospreys and also measured the concentrations of a range of pharmaceuticals in water, prey fish and nestling osprey plasma to confirm the validity of the model. Of 18 pharmaceuticals detected in water, only the antihypertensive diltiazem was detected in nestling plasma; levels were below the human therapeutic concentrations in all 47 nestlings that were analysed. Studies on another freshwater sentinel, the Eurasian otter (*Lutra lutra*), indicates that oral and/or dermal exposure to diclofenac and ibuprofen is taking place in the UK [71]. Renal lesions observed during carcass necropsies have prompted recommendations that future studies examine exposure of otters to nephrotoxic agents such as NSAIDs [72]. In general, top predators are likely to be most susceptible to pharmaceuticals that bioaccumulate and bioconcentrate in prey.

### (b) Impacts on higher vertebrates associated with terrestrial systems

The clearest example of widespread exposure and effect relates to the impact of the NSAID diclofenac on vultures, as described in detail in this special issue [18]. Carcass dumps are now well recognized as a critical exposure pathway for pharmaceuticals to vultures in Asia [18], and there are current similar concerns about the susceptibility of scavengers (vultures and kites) to antibiotics (and to NSAIDs and antiparasitics) when feeding at muladares (small carcass dumps) in Spain. There have been some studies that indicate the occurrence of various adverse effects on egg development, immunocompetence and disease prevalence, but all relevant data regarding these Spanish carcass dumps have now been retracted from publication. As such, the real picture in Spain specifically remains unclear. Further investigation of the potential for exposure and effects of pharmaceuticals in vultures in Spain (a very important stronghold for vultures in Europe) and in Africa is merited. Studies in Spain are particularly warranted given: (i) the 2013 approval of diclofenac for veterinary use in that country [73], (ii) the fact that some carcasses provided at muladares, managed feeding stations and at captive breeding and rehabilitation centres, are known to originate from intensive operations such as large pig rearing facilities where animals are heavily medicated, and (iii) a concern that risk assessments seem poorly developed for scavengers, as already highlighted in §3.

Antiparasitics are also widely used on livestock making them, in effect, pharmaceuticals. Many, such as organophosphates, carbamates and pyrethroids, were developed as agrochemicals and so, unlike most pharmaceuticals, their adverse effects in non-target wildlife are relatively well described. Antiparasitic applications of famphur, fenthion, diazinon and propetamphos to lambs, cattle and pigs have been linked to bird of prey poisonings in Canada, USA and the UK [74]. Because of concerns about infectious diseases such as foot and mouth disease virus, carcasses of small domestic ruminants are no longer left to be ‘incorporated into nature’, and slaughterhouse remains are meant to provide safe food sources for vultures. Lower limbs of lambs collected from a Spanish slaughterhouse have been shown to contain up to 618 ng g^−1^ of diazinon and 1008 ng g^−1^ of cypermethrin [52]. These portions of carcasses are currently being offered at vulture feeding stations in the Pyrenees. Although the estimated dose of diazinon was considered below the acute avian LD_50_ for these compounds, chronic effects (induced hypothermia or behavioural impairment [75]) cannot be ruled out.

Scavengers can also be poisoned by feeding on euthanized animals. Residues of barbiturates in carrion have been found to exceed the lethal dose for a spectrum of scavengers [76,77], and there have been reports of secondary barbiturate poisoning [78]. In some parts of the USA, landfills are legal areas for dumping the carcasses of euthanized animals, and free living bald eagles (*Haliaeetus leucocephalus*) and golden eagles (*Aquila chrysaetos*) are known to have died of barbiturate poisoning; in northern climates, late winter—early spring has been the most common time to find poisoned raptors and scavengers as carcasses are thawing during a time of low food availability [79]. While poisoning of large-bodied animals in areas with moderately high human populations is likely to be detected, as on Vancouver Island (Canada), where 29 bald eagles were intoxicated after feeding on a euthanized (sodium pentobarbital) cow [55], incidents in more remote areas may not be detected. Furthermore, incidents involving smaller, less conspicuous animals are more likely to go unnoticed, as is evident from mortality incident monitoring schemes; for example, submissions of mammals to the UK Wildlife Incident Investigation Scheme are dominated by large species like red fox (*Vulpes vulpes*) and badger (*Meles meles*) [80]. Such biases mask the true extent of exposure and poisoning of wildlife by PPPs, pharmaceuticals or any other toxicant.

There is also a notable dearth of information regarding risks to terrestrial wildlife from diffuse pharmaceutical sources, such as fertilization of agricultural land with slurry and biosolids. Studies on agricultural animals suggest potential exists for effects. Testosterone and oestrogen, used as growth promoters in chicken production have, for example, caused endocrine disruption in cattle fed rations containing chicken manure. One-quarter to one-third of heifers fed chicken manure silage did not reach puberty and animals already past puberty developed cystic ovaries and stopped cycling after one month of exposure [81]. The likelihood of such effects occurring in wildlife would depend, among other things, on how much feeding took place in a fertilized area and the persistence of pharmaceuticals in soil. The one specific study that we are aware of has again only been reported to date as a conference abstract [82]. In a seven year experimental study in which triclocarben- and triclosan-contaminated biosolids were applied to fields, concentrations of these two antimicrobials were measured in biosolids, soils, earthworms and the eggs of American kestrels (*Falco sparverius*) and European starlings (*S. vulgaris*). Kestrel and starling egg morphometrics and nesting success were also tracked. The antimicrobials were detected in biosolids, soil, earthworms and the eggs of both avian species. Concentrations were higher in samples from the experimental than the control site for starling eggs, soil and worms, but not kestrel eggs. Nesting success was lower on the experimental than control site for kestrels but not starlings. This study provides evidence that antimicrobials from biosolids can be transferred to eggs of secondary and tertiary consumers and begs more research on the food-chain transfer and biological impacts of pharmaceuticals in terrestrial higher vertebrates.

## 5. How are exposure and effects detected in wildlife for other contaminants of concern?

Once released into natural habitats, pharmaceuticals can be considered just another class of environmental contaminant. While few monitoring studies have examined exposure and effects of pharmaceuticals in higher wild vertebrates, various programmes currently investigate uptake and effects of toxic metals, pesticides, biocides and organic pollutants in higher vertebrates. Some primarily measure exposure (and limited effects), whereas others are specifically tailored to determine effects, typically mortality, and their cause [83–86].

A number of schemes that focus on vertebrate exposure to pollutants also measure contaminants in dietary items. This approach forms the basis for regulatory assessment of risk, as described in §3, but there are challenges in terms of detecting and measuring exposure to diffuse pollutants. This is because environmental concentrations of such compounds may often be low with typically high spatio-temporal variability [87]. An alternative is to measure contaminants in sentinel species that are representative of particular trophic pathways. Such species are often apex predators that can bioaccumulate, and in some cases bioconcentrate, pollutants. Tissue concentrations then rise above analytical detection limits and this can be critical in terms of clearly identifying the presence and effects of contaminants within a system. A range of predatory birds and mammals have been used as sentinels [84,88,89]. Typically long-lived species with large foraging ranges, sentinel species can integrate exposure both temporally and spatially, thereby smoothing small-scale variability and aiding detection of large-scale and long-term trends. Furthermore, large vertebrate predators are often seen as charismatic species. Data regarding such species tend to excite public and stakeholder interest, and this can be important when the discovery and collection of dead wildlife relies on citizen science, hunters or other representative groups within a community [90–92].

The exposure of higher vertebrates to a range of contaminants has typically been demonstrated using a residue monitoring approach. Tissue residues are by definition indicative of the bioavailable fraction of the environmental concentration and the favoured types of samples include various body tissues, blood, eggs, hair, feathers and other substances such as preen oil [84,88–91]. Exposure studies can be critical in identifying key factors that drive exposure [93–96], can relate measured residues to levels known to cause adverse effects [97], and measure effects directly using biomarkers [98–101]. We are not aware of any current long-term wildlife monitoring schemes that target pharmaceutical residue analysis, though pleas have been extended [23,48].

Some national monitoring schemes have an effects-based approach, particularly regarding poisoning [83,85]. Such mortality incident monitoring schemes are intended to detect significant adverse effects, ostensibly mortality, caused by authorized PPPs and, in some cases, other approved toxicants. Effects brought to light by such schemes would often not have been foreseen during the risk assessment and authorization process and their occurrence in turn may trigger review of authorizations and amendments to risk assessment processes. Such schemes are also used to detect and investigate the misuse and illegal abuse of pesticides and have, for example, identified significant predatory bird and mammal mortality owing to a wide range of poisons, chemicals, pesticides and biocides [74,85,102].

The key question here is whether exposure and mortality incident monitoring schemes can be used to accurately consider exposure/effects of pharmaceuticals in wild higher vertebrates, or are bespoke stratagems needed? Detection of mortality alone is relatively crude and, as indicated in §4, pharmaceuticals may well induce more subtle changes in exposed wildlife. Given the general absence of data, determining the current prevalence and extent of exposure in wildlife would arguably be the first priority. Monitoring schemes that rely upon apex predators as sentinels may best detect exposure to pharmaceuticals that have high bioaccumulation potential. Such schemes should perhaps focus pharmaceutical monitoring on species that are insectivorous and/or vermivorous in terrestrial systems, piscivorous in freshwater habitats or general scavengers as these three pathways are the likely major exposure routes; this would also be compatible with monitoring for pesticide exposure [74]. Development of techniques to measure trace residues of pharmaceuticals in non-destructive samples, such as feathers, hair, wool and faecal matter [43,48,54,71] can facilitate monitoring without capture of, or disturbance to, animals, and may be an ideal means to detect exposure to pharmaceuticals that have short half-lives and would be unlikely to be detected in physiologically active tissues. For example, feathers and wool can be gathered from nest sites and hair can be collected using hair tubes (small mammals) and hair-snagging stations (large mammals). Such samples can be analysed for DNA (to distinguish species/individuals) and chemical residues. These techniques may be particularly important when species are secretive and hard to capture, and can be used to gather large numbers of samples at relatively low cost. They are already being used to monitor exposure and, in some cases, effects associated with pesticides and pollutants [84,103] and are likely to be equally useful for monitoring pharmaceuticals [48].

## 6. Discussion

It is often difficult to predict the complete life cycle of a chemical in the environment at a national or regional scale because of economic, social and cultural differences. However, if pharmaceuticals are to be developed for use in a safe manner, the possibility that they may reach non-target wild higher vertebrates should be considered. In the European Union (EU) for example, active PPP ingredients are authorized by a pan-EU body (the European Food Safety Authority), but product authorizations and associated risk mitigation strategies remain the preserve of national entities. Such an approach can incorporate regional life cycle assessments and risks factors and implement associated mitigation. Regulations for pharmaceuticals should work in a similar manner.

The impact of diclofenac on vultures in Asia in particular has demonstrated the need for national or regional ecopharmacovigilance strategies to support regulatory risk assessment. The existence of exposure and mortality incident monitoring schemes in some regions, such as Western Europe and North America, provides a good platform for ecopharmacovigilance, although programmes are needed elsewhere. Information on the national/regional scale of pharmaceutical use is required if monitoring schemes are to establish adequate analytical screening methods. These should be linked to modelling approaches that are designed to identify which compounds/classes of compounds are most likely to persist in the environment and bioaccumulate, thereby facilitating the targeting of analytical effort. Exposure monitoring schemes that use apex predators will be best suited to monitoring less polar, more persistent compounds, but may need to include additional sentinel species that scavenge or feed on invertebrates, the latter representing an exposure pathway that is relatively poorly covered by existing schemes [84]. In contrast to exposure monitoring programmes, mortality incident monitoring schemes usually examine mortalities in a wider range of species, including those from lower trophic levels. Although we have argued that mortality is a crude means of assessing any impact of pharmaceuticals, mortality monitoring schemes could facilitate ecopharmacovigilance by greater use of the carcasses of animals they receive. This would require a widening of remit, so that schemes undertook (or made carcasses available for) screening of samples to determine if there has been exposure to pharmaceuticals; currently, analyses are often limited to compounds suspected to be the cause of death and wider analyses are not undertaken. Such engagement, particularly using species from lower trophic levels, would be especially useful for investigating exposure (and possibly pinpointing inputs) to less persistent and less bioaccumulative compounds that are unlikely to be detected by monitoring schemes using apex predators. Successful national and regional ecopharmacovigilance would best be served by coordination between different schemes, even though they may have different aims and stakeholder perspectives. Models to improve collaboration and knowledge exchange between diverse exposure and mortality monitoring schemes already exist and have proven successful [104].

This review has also highlighted that, while existing generic risk assessment procedures for chemicals should be applicable to pharmaceuticals, major modifications may be necessary. Exposure scenarios may not adequately assess certain critical pathways, such as those applicable to scavengers. The toxicity endpoints, and in some cases, the test species, currently used in risk assessment are also unlikely to detect the more subtle chronic effects that pharmaceuticals may exert on wildlife. Developing new sensitive tests and endpoints, and demonstrating their value for assessing likely impacts on populations, is challenging, but such work would benefit risk assessment not only for pharmaceuticals, but also PPPs and other environmental contaminants generally.

A slightly tangential, but highly relevant issue related to drug exposure in higher wildlife is that of exposure to antibiotics, antibiotic-resistant genes and multi-drug-resistant microbes. Antibiotic-rich effluents are of particular concern as they have been shown to promote the development of antibiotic-resistant microbes in the environment, which could have unpredictable and wide reaching consequences for human and wildlife health [15,105]. Antimicrobial drug use is also especially common in intensive livestock operations, including CAFOs [38,44], and residues may enter the terrestrial environment by various routes. Furthermore, inhalation of CAFO aerosols may facilitate the transfer of multi-drug-resistant bacterial pathogens from farmed animals to exposed humans [42,106], highlighting the potential importance of inhalation pathways, which have not previously been considered *vis a vis* the impacts of pharmaceuticals on the environment. Overall, the primary concern regarding antibiotics is not so much direct effects on wildlife, but rather that wildlife could play a role in the transfer of antibiotic-resistant microbes between humans and the environment [105–110]. While worthy of note, a comprehensive review of this complex, and likely increasingly important, issue is outside the scope of this paper.

In conclusion, the increasing use of medication by a burgeoning human population and its agricultural livestock ensures an ongoing and likely increasing release of pharmaceuticals to the environment. This, coupled with other pressures, such as human demographic change leading to the development of super-cities, increased intensification of food production and climate-induced hydrological changes, may alter our ability to limit, regulate and dilute pharmaceutical discharges to the environment [111]. It is notable that two of the most important wildlife ecotoxicological cases recently associated with environmental pollutants (widespread vulture declines and feminization of fish) are directly related to the use and disposal of pharmaceuticals rather than to other drivers. Existing frameworks and platforms can be used to help us improve our knowledge of current and future environmental concentrations and effects of pharmaceuticals. The development and application of that knowledge is now a priority.
